# Health Resource and Cost Savings Achieved in a Multidisciplinary Lung Cancer Clinic

**DOI:** 10.3390/curroncol28030157

**Published:** 2021-04-29

**Authors:** Christopher J. L. Stone, Ana P. Johnson, Danielle Robinson, Andriy Katyukha, Rylan Egan, Sophia Linton, Christopher Parker, Andrew Robinson, Geneviève C. Digby

**Affiliations:** 1 Department of Medicine, University of Ottawa, Ottawa, ON K1H 8L6, Canada; 12cjls@queensu.ca; 2 Department of Public Health Science, Queen’s University, Kingston, ON K7L 3N6, Canada; ana.johnson@queensu.ca; 3 School of Medicine, Queen’s University, Kingston, ON K7L 3N6, Canada; drobinson@qmed.ca (D.R.); a.katyukha@queensu.ca (A.K.); 4 School of Nursing, Queen’s University, Kingston, ON K7L 3N6, Canada; rylan.egan@queensu.ca; 5 Department of Medicine, Queen’s University, Kingston, ON K7L 5P9, Canada; 18sml12@queensu.ca (S.L.); parkerc@queensu.ca (C.P.); 6 Department of Oncology, Queen’s University, Kingston, ON K7L 5P9, Canada; andrew.robinson@kingstonhsc.ca

**Keywords:** lung cancer, quality improvement, resource utilization, patient care, health economics

## Abstract

**Background**: Lung cancer (LC) care is resource and cost intensive. We launched a Multidisciplinary LC Clinic (MDC), where patients with a new LC diagnosis received concurrent oncology consultation, resulting in improved time to LC assessment and treatment. Here, we evaluate the impact of MDC on health resource utilization, patient and caregiver costs, and secondary patient benefits. **Methods**: We retrospectively analyzed patients in a rapid assessment clinic with a new LC diagnosis pre-MDC (September 2016–February 2017) and post-MDC implementation (February 2017–December 2018). Data are reported as means; unpaired t-tests and ANOVA were used to assess for significance. We also conducted a cost analysis. Resource utilization, out-of-pocket costs, procedure-related costs, and indirect costs were evaluated from the societal perspective and presented in 2019 Canadian dollars (CAD); multi-way worst/best case and threshold sensitivity analyses were conducted. **Results**: We reviewed 428 patients (78 traditional model, 350 MDC). Patients in the MDC model required significantly fewer oncology visits from LC diagnosis to first LC treatment (1.62 vs. 2.68, *p* < 0.001), which was significant for patients with stage 1, 3, and 4 disease. Compared with the traditional model, there was no change in mean biopsies/patient (1.32 traditional vs. 1.17 MDC, *p* = 0.18) or staging investigations/patient (2.24 traditional vs. 2.02 MDC, *p* = 0.20). Post-MDC, there was an increase in invasive mediastinal staging for patients with stage 2/3 LC (15.0% vs. 60.0%, *p* < 0.001). Over 22 months, MDC resulted in savings of CAD 48,389 including CAD 24,167 CAD in direct patient out-of-pocket expenses. For the threshold analyses, MDC was estimated to cost CAD 25,708 per quality-adjusted life year (QALY), considered to be below current willingness to pay thresholds (at CAD 80,000 per QALY). MDC also facilitated oncology assessment for 29 non-LC patients. **Conclusions**: An MDC led to a reduction in patient visits and direct patient and caregiver costs.

## 1. Introduction

Lung cancer (LC) is the leading cause of cancer-related mortality [1] and is associated with significant health system resource utilization [2,3,4,5]. Timely LC care requires the integration of various specialists, given the diverse management options, frequently advanced presentation, and disparities in care [6]. Delivering timely LC care is challenging, with delays in diagnosis and treatment being common [7]. These delays can lead to tumour progression [8] and patient distress [9]. In turn, LC care is becoming increasingly expensive for patients, caregivers, and the healthcare system, with costs related to provider visits, investigations, and new treatment modalities rising over the past decade [2,10].

Multidisciplinary cancer clinics (MDC) have increasingly been shown to have several benefits in LC care, including: improved care coordination, communication between providers, and compliance with guideline-recommended care; reduced delays in time to diagnosis and treatment; and reduced healthcare costs from improved resource utilization, fewer patient visits, and reduced out-of-pocket costs for patients [3,4,5,11,12,13,14,15]. Furthermore, there is evidence that involvement of a Respirologist in the care of LC patients has been shown to improve patient quality of life measures and survival through optimization of obstructive lung disease [16,17], highlighting the role of comprehensive patient evaluation and optimization prior LC treatment. That said, there have been no studies to date that assess the clinical efficiencies achieved through quality improvement (QI) in an LC MDC, and few studies evaluating the health resource utilization and secondary patient benefits achieved through LC MDC models [3].

Kingston Health Sciences Centre (KHSC) is an academic tertiary care centre that serves a catchment area of greater than 500,000 residents living in a 20,000-square-kilometre, predominantly rural area. The Lung Diagnostic Assessment Program (LDAP) at KHSC is a rapid assessment clinic responsible for coordinating care for patients with suspected LC, through which approximately two-thirds of the region’s LC diagnoses are made.

To streamline patient diagnostic and treatment pathways, an LC MDC was implemented in the LDAP in 2017 and has since undergone continuous QI initiatives to improve clinical efficiencies and timeliness of care. Within a year of implementation, the LC MDC led to decreased time from LC diagnosis to oncology assessment of 10 days and decreased time to first treatment of 25 days [18]. Qualitative evaluation of patient, caregiver, and provider perceptions of the clinic revealed positive experiences and perceived benefits by all clinic participants [14]. Now, more than two years following MDC implementation, we seek to characterize the impact of QI on MDC clinic capacity, resource utilization, patient and clinic visit costs, and secondary patient benefits.

## 2. Patients and Methods

### 2.1. Context—Launching a Lung Cancer MDC

The LDAP at KHSC consists of parallel Respirology and Thoracic Surgery streams, to which patients are triaged according to suspicion of resectable and operable LC based on available clinical information and imaging at the time of triage. Historically at KHSC, the majority of patients (75–80%) have been triaged to the Respirology stream for assessment. Once a cancer diagnosis was confirmed in either LDAP stream, patients returned to the clinic to obtain biopsy results, and outpatient referrals to oncology were subsequently sent. Our initial quality improvement initiative sought to improve care for patients in the Respirology LDAP stream.

In February 2017, we implemented a weekly LC MDC in the Respirology stream involving Respirologists, Medical Oncologists (MO), and Radiation Oncologists (RO) (≥1 physician per specialty, per clinic), where Respirologist-managed LDAP patients with a new LC diagnosis were offered concurrent oncology consultation at the time of diagnosis disclosure. At every MDC, physicians met to pre-review patients booked in the clinic, discuss the need for oncology consultation, and develop preliminary management plans. Each oncology visit in the MDC could include up to three individual physician interactions, with any/each of the aforementioned specialists. Plan–Do–Study–Act (PDSA) cycles were used to guide MDC implementation and continuous clinic QI, as outlined in Stone et al. [18], and occurred in response to identified areas for improvement based on real-time needs assessment.

In short, in the first year of clinic launch, PDSA cycles and improvement interventions included: a pilot MDC to establish feasibility (July–August 2016), large-scale MDC launch (February 2017), stakeholder debriefs and retreats to troubleshoot inefficiencies (April 2017), and adding additional clinics to address increasing patient referral volumes (September 2017). Since October 2017, further improvement efforts have included: standardization of oncologist availability in MDC to ensure regular representation of all specialists listed above (October 2017), improved cross-coverage of the clinic by all specialists (December 2017), increased frequency of participation of Thoracic Surgery (2018 onwards), and streamlining transitions of care to the regional Cancer Centre for initiation of LC treatment(s) (May 2018).

### 2.2. Evaluation of the LC MDC

We retrospectively analysed Respirologist-managed LC patients seen in the traditional LDAP model to establish baseline data [18], with data collected used for this second study. We prospectively analysed all MDC patients seen from February 2017 to December 2018 and collected data regarding (i) patient characteristics: age, pathologic cancer diagnosis, LC stage, and distance lived from KHSC; (ii) timeliness of care measures: date of diagnosis (pathologic confirmation or decision to treat based on suspicious imaging when biopsy contraindicated), date of oncology assessment, date of first cancer treatment including treatment type (thoracic surgery (TS), radiation (RT) or systemic therapy (ST)), and (iii) health system resource utilization (number of oncology visits) and clinic visit costs.

For patients from September 2016 to October 2017, we also collected data regarding the number and type of staging imaging, number and type of tissue biopsies for diagnosis and/or staging (e.g., endobronchial ultrasound transbronchial needle aspiration (EBUS-TBNA), CT-guided biopsy, or percutaneous biopsy of metastatic site). This more limited time frame for evaluation of number and types of staging tests was due to a combination of availability of data and introduction of concurrent improvement processes seeking to standardize the ordering of staging investigations [19], which we felt could be a confounder in the data analysis if further data were to be included.

Descriptive data are reported as *n* (%) and means. Unpaired t-tests, chi-squared, and ANOVA were used to assess for pre versus post-MDC significance. The primary analysis included patients with non-small-cell (NSCLC) and small-cell lung cancer (SCLC). We performed subgroup analyses according to type of cancer treatment and clinical LC stage for health resource utilization and secondary patient benefits [20].

### 2.3. Economic Analysis

The economic evaluation used standard techniques for cost analysis [21,22] and adopted a societal perspective—all costs were included in the analysis, regardless of who paid or benefitted—to estimate savings. All costs were expressed in 2019 Canadian dollars (CAD) [23]. The cost analysis identified administrative resources, out-of-pocket expenses, and change in productivity by determining the per visit cost associated with these variables and calculating total cost savings as a result of MDC implementation. Specifically, we (i) categorized resources, (ii) tracked resources in specific units, (iii) calculated the cost per unit for each resource type, presented the number of units consumed in each resource type, and expressed this in terms of costs per patient and costs overall, and (iv) calculated cost savings as a result of MDC. MDC cost savings were subdivided into categories: (i) clinic visit costs as a result of reduced patent visit bookings; (ii) out-of-pocket expenses from travel and parking (based on clinic visit time, travel time, parking time, and time locating clinic); and (iii) opportunity costs associated with travel and parking (patient charts were reviewed to estimate the number of caregivers per patient on average for clinic visits). No additional personnel costs were incurred to carry out the quality improvement initiatives or evaluation.

Multi-way (“best case” and “worst case”) and threshold sensitivity analyses were conducted, altering clinic visit duration based on available time ranges published in the literature, parking cost (paying for full hour versus partial hour during clinic visit), and productivity loss in order to evaluative the robustness of the results. For the threshold sensitivity analysis, estimated cost-effectiveness was compared with current willingness to pay thresholds per quality of years of life, predominantly at CAD 80,000 per quality-adjusted life year (QALYs). Variables, cost estimates, and sensitivity analyses are detailed in the Supplementary Materials.

### 2.4. Invasive Mediastinal Staging by EBUS Economic Analysis

The economic evaluation included multi-way and threshold sensitivity analyses evaluating the costs associated with invasive mediastinal staging by EBUS-TBNA for the subgroup of stage 2/3 LC patients for whom invasive mediastinal staging is recommended [24]. The analysis identified procedural costs, out-of-pocket expenses, and change in productivity by determining per procedure cost associated with these variables and estimating the total QALYs added as a result of invasive mediastinal staging. This information was then compared to published willingness to pay thresholds (how much a decision-maker is willing to pay for a program) to establish the cost-effectiveness of the intervention, given a significant estimated increase in the use of EBUS-TBNA for patients in the MDC. Invasive mediastinal staging costs were subdivided into categories: (i) procedure costs associated with EBUS-TBNA including procedural sedation; (ii) out-of-pocket expenses from travel and parking (based on visit time, travel time, parking time, and time locating the endoscopy unit); and (iii) opportunity costs associated with travel and parking, as outlined above.

Our study was approved by the Queen’s University Health Sciences and Affiliated Teaching Hospitals Research Ethics Board (DMED-2002-17).

## 3. Results

### 3.1. Clinic Characteristics

Data were collected from 78 patients prior to MDC launch (traditional model) to establish a baseline period of stability (September 2016 to February 2017) from which improvement could be assessed. Since MDC launch in February 2017, 350 patients have been seen in 102 clinics (average of 3.4 patients/clinic). The number (*n*) of patients seen per MDC increased from 2.3 patients/clinic in February 2017 to 4.0 patients/clinic in August 2018. This subsequently increased to a maximum of 6.0 patients/clinic in December 2018 in response to increased intake of non-LDAP managed patients and identification of patients that would benefit from multidisciplinary care.

The mean age of patients in the traditional model was 70.0 ± 9.7 years and in the MDC model was 71.7 ± 9.1 years (Table 1). Of baseline patients, 69 (88.5%) had NSCLC and 9 (11.5%) had SCLC. In the MDC model, 260 (74.3%) had NSCLC, 45 (12.9%) had SCLC and 45 (12.9%) had a clinical LC diagnosis without biopsy but were included in the analysis as they received concurrent oncology consultation.

### 3.2. Impact on Number of Oncology Visits

Compared to baseline, patients seen in the MDC model required significantly fewer oncology visits from date of diagnosis to first cancer treatment (1.62 vs. 2.68, *p* < 0.001) (Table 2). There was a reduction in visits for all stages of LC, with a significant reduction for patients with stage 1, 3, and 4 disease, and underpowered to assess significance for patients with stage 2 disease (*n* = 32).

### 3.3. Impact on Biopsies, Staging Investigations, and Treatment

Data regarding biopsies and staging tests were collected from September 2016 to October 2017. Compared with baseline, there was no significant difference in the mean number of biopsy procedures for diagnosis and/or staging in the MDC (1.32 at baseline vs. 1.17 in MDC, *p* = 0.18). There was no significant difference in the mean number of staging imaging per patient (2.24 at baseline vs. 2.02 in MDC, *p* = 0.20). The number of patients who underwent guideline-recommended mediastinal staging [25] by EBUS-TBNA for stage 2 and 3 LC increased significantly from 15.0% at baseline to 60.0% in MDC (*p* < 0.001).

MDC did not significantly impact the treatment of patients with LC (Table 2). The percentage of patients with stage 1 or 2 LC who received curative intent therapy (TS or RT) was unchanged between the traditional model and MDC (86.7% vs. 88.1%, *p* = 0.83). The percentage of patients with Stage 3 NSCLC who received curative intent therapy did not change significantly (57.1% vs. 53.6%, *p* = 0.82). The percentage of patients with stage 4 LC who received ST did not differ between baseline and in the MDC model (37.9% vs. 39.9%, *p* = 0.85).

### 3.4. Economic Analysis

The economic analysis entailed the calculation of cost savings attributed to an average of 1.06 visits/patient saved from MDC implementation, for an estimated 371 visits saved (1.06 visits/MDC patient × 350 MDC patients). Ultimately, MDC resulted in savings of CAD 48,389 or CAD 138/patient seen, during the study period (Table 3). Extrapolating these cost savings to an estimated 585 patients seen in MDC over the first 3 years of the program (February 2017–February 2020), the overall cost savings are estimated at CAD 80,882.

Clinic visit cost was calculated from administrative personnel hourly wages (Supplementary Materials) at an estimated 3 min required to schedule each visit. Given the 371 visits saved, net administrative savings was CAD 508.

Out-of-pocket expenses included the cost of return travel and hospital parking at clinic visits. Travel cost was estimated based on average return travel distance from MDC patients’ home to the clinic at the travel allowance rate of CAD 0.58/km [26], for each clinic visit. Parking costs were calculated from average parking costs in at KHSC at CAD 3/hour [27]. Given the 371 patient visits saved in MDC, the total out-of-pocket savings in MDC for all patients studied (*n* = 350) was CAD 24,167 or CAD 69/patient.

Productivity loss was calculated based on an income loss of CAD 29.55/hour [28] over the time calculated for an average clinic visit [29], return travel time, and estimate of time for parking and finding the clinic (2.53 h). Productivity loss was only evaluated for patients <65 years old, comprising 23% of MDC patients (79/350) [30,31]. Given 371 fewer visits in MDC, this resulted in patient productivity loss reduction of CAD 6,379. Furthermore, through examining patients’ charts, we found that, on average, there were approximately 1.25 (range 1–4) caregivers per patient present at each visit. In our base case scenario, we estimated that 50% of caregivers incurred productivity loss, based on previously published values [10,32,33], due to clinic visit attendance. Productivity loss associated with caregivers’ time was estimated using the aforementioned time variables, resulting in a savings of CAD 17,335.

The cost analysis was moderately sensitive to changes in productivity loss [29,34] (best case 55.5 min clinic visit [34], with 25% of caregivers losing work [35]; worst case, 94.8 min clinic visit [36], 7.5 h off work for both patient and caregivers, 75% of caregivers losing work [33,37]), parking (best case: CAD 4.50; worst case: CAD 12); leading to patient and clinic visit economic benefits, with a total cost reduction range of CAD 37,173–122,896 (Supplementary Materials).

### 3.5. Threshold Sensitivity Analysis—Invasive Mediastinal Staging by EBUS-TBNA

Given that the number of patients who underwent guideline-recommended mediastinal staging [25] by EBUS-TBNA for stage 2 and 3 LC increased significantly from 15.0% at baseline to 60.0% in MDC, we performed a threshold sensitivity analysis taking into consideration costs and quality of life years attributed to EBUS-TBNA in order to assess the cost-effectiveness of the program, given decision-makers’ willingness to pay thresholds [38]. The cost per EBUS-TBNA procedure in 2019 CAD was estimated to be CAD 1577, which falls within published cost ranges of CAD 1187 to 4370 [38,39,40]. Ultimately, EBUS-TBNA procedure costs totalled CAD 4731 in the traditional model (3 EBUS-TBNA performed for 20 patients) and CAD 89,889 in the MDC model (57 EBUS-TBNA performed for 95 patients). QALY was calculated as 0.071 to represent the measure of quality of life associated per EBUS-TBNA [38], referenced from a study that best approximated our local context; the only other reported QALY value for EBUS-TBNA available was 0.015 [41], from a European study, which was less representative of our local context. Willingness to pay thresholds for EBUS-TBNA vary between CAD 45,000 [42] and 148,000 [43]. Here, CAD 80,000 per QALY was considered [38] as it best approximates our context, with EBUS-TBNA provided under conscious sedation within the same single-payer healthcare system and province.

Out-of-pocket expenses included the estimated cost of return travel and hospital parking for the EBUS-TBNA procedure. Travel costs were estimated as above. Parking costs were calculated from average KHSC parking costs at CAD 3/h [27], with an estimate of 3 h parking per procedure, and 50% of patients requiring parking during the procedure. This resulted in a cost of CAD 192 and 3627 in the traditional and MDC models, respectively. Productivity loss was calculated based on an income loss as above, however we noted that patients would require a full day away from work (7.5 h) given the procedure and recovery times. Productivity loss was only evaluated for patients <65 years old, comprising 33% of patients in the traditional (1/3 patients) and MDC model (19/57 patients). This resulted in patient productivity losses of CAD 221 and 4207 in the traditional and MDC models, respectively. Productivity loss associated with caregivers’ time was estimated using the aforementioned time variables, resulting in costs of CAD 333 and 6316 in the traditional and MDC models, respectively. Recognizing that each patient required a caregiver for transportation home post procedure, we estimated that 50% of these caregivers incurred productivity loss, based on previously published values [10,32,33].

The threshold sensitivity analysis showed that for invasive mediastinal staging by EBUS-TBNA to be cost saving (at a willingness to pay threshold of up to CAD 80,000: (COST_MDC_ − COST_Trad_)/(QALY_MDC_ − QALY_Trad_) < CAD 80,000), we would need to see a difference of at least 1.23 QALYs when comparing MDC with the traditional model: QALY_MDC_ − QALY_Trad_ = CAD 80,000/(COST_MDC_ − COST_Trad_) = CAD 80,000/(CAD 104,039 − CAD 5476) = 1.23. As noted in Table 4, an estimated difference of 3.84 QALY was observed in MDC (QALY_MDC_ − QALY_Trad_ = 4.05–0.21 = 3.84). Likewise, when comparing MDC with the traditional model, EBUS-TBNA was estimated to cost CAD 25,708 per QALY, which falls below the willingness to pay threshold of CAD 80,000 per QALY.

### 3.6. Secondary Unanticipated Benefits

The MDC clinic was designed to facilitate oncology assessment for Respirology LDAP-managed patients returning to receive results of diagnostic and staging investigations. As the clinic evolved, the potential for early oncologic assessment for patients with presumed LC (prior to pathologic confirmation) with symptomatic metastases became apparent. In the baseline period, these patients were referred to see an oncologist in the Cancer Centre or admitted to hospital for symptomatic disease management. Following MDC launch, 31 patients seen in the MDC (8.9%) were seen by an oncologist at first LDAP visit prior to tissue diagnosis. Of these, 26 saw Radiation Oncology (RO), 1 saw Medical Oncology (MO), and 4 saw both MO and RO. Furthermore, 20 (64.5%) of these patients went on to receive treatment with a mean time from diagnosis to treatment of 5.3 days (compared with the average time to treatment of 33 days for all patients in the MDC model), and with 4 patients (12.9%) receiving RT before tissue diagnosis for urgent indications.

We observed that 29 patients returning to MDC to receive a non-LC diagnosis were able to receive concurrent oncology consultation when an oncologist in clinic treated that disease site. Four were patients with mesothelioma who were also seen in MDC but were not included in the primary analysis, which only included patients with NSCLC and SCLC. The other 20 non-LC malignancies included 8 patients with breast cancer; 2 patients with each of lymphoma, thymic, and urothelial cancer; and 1 patient with each of hemangiopericytoma, neuroendocrine not otherwise specified, ovarian, myeloma, leiomyosarcoma, thyroid. Five patients had oncology consultation that went on to have biopsy proven benign lesions (hamartoma and granuloma).

### 3.7. Balancing Measures

We found no difference in mean number of patient visits between referral for evaluation and diagnosis in LDAP at baseline (1.24 visits/patient) compared with MDC (1.26 visits/patient), *p* = 0.89. Overall, in the post-MDC period, 42 (12.0%) patients with a new LC diagnosis who were not seen in the MDC, for reasons including: 10 patients brought back to a non-MDC clinic (either Respirology or Oncology clinic); 10 patients with stage 1 LC referred directly for surgical opinion and/or management; 9 patients who declined oncology assessment, and 9 patients who required hospital admission and received oncology consultation as an inpatient. Observed improvements did not affect time commitments as physicians saw the same number of patients in the reorganized clinic structure. This is consistent with our previous findings that the proportion of patients receiving Oncology consultation and treatments did not vary between the traditional model and the MDC model as a whole, nor by paired analysis by LC stage [18].

## 4. Discussion

Implementation and continuous QI of an LC MDC clinic led to improved clinic capacity and health resource utilization, patient and clinic visit cost savings, and secondary patient benefits. MDC clinic capacity increased while maintaining efficiency, reduced individual patient oncology visits prior to first cancer treatment, facilitated non-LC patient care, and reduced administrative, patient, and caregiver costs. Previously published data from our MDC also demonstrated a 10-day decrease in time from LC diagnosis to oncology assessment and a 25-day decrease in time from LC diagnosis to first cancer treatment [18] and also led to patient-reported benefits of satisfaction with care, convenience, and positive effect of family presence at appointments [14]. While the evidence for MDC models is limited outside of our clinic model [11,44], MDCs have been shown to improve the timeliness of care, staging and treatment guideline compliance, and reduce patient distress levels [11,13,45,46,47,48,49]. We build on these findings by demonstrating that QI in healthcare can create sustainable change [50,51,52], improve efficiency for patients and the healthcare system, and can lead to unanticipated benefits including improved adherence to guideline recommended mediastinal staging. While the economic evaluation is focused on the direct impact of the MDC clinic, and may not provide a comprehensive evaluation of the entirety of the costs associated with LC care, we include an assessment of potential increased costs associated with increased invasive mediastinal staging.

The number of patients seen in MDC increased over time as a result of implemented measures to maintain timeliness of care, including the inclusion of a community MO in the clinic rotation and cross coverage of Respirologists and Oncology specialists to ensure no cancelled clinics. Sustainability and spread are key markers of successful QI and maintenance of gains from an improvement project [52]; as such, these changes were essential to ensure the ongoing success of our program. Furthermore, sustainability does not happen by chance [52]; providers reported perceived improved communication and collegiality, clinic efficiency, and patient outcomes [14], which created a culture of improvement and sustainability in MDC.

MDC led to a statistically significant reduction in the number of oncology visits between diagnosis and first cancer treatment (2.68 to 1.62), with subgroup analysis demonstrating significance for patients with stages 1, 3, and 4 disease. Voong et al. found there was a significant reduction of two provider visits per patient (6.8 versus 4.8) from referral to treatment [3] in MDC; however, each individual clinician interaction was counted as one visit. In contrast, in our MDC, a visit included up to three individual clinician interactions which was counted as one individual oncology visit. This reduction in oncology visits likely contributed to improved time to LC treatment, as demonstrated previously [18]. Although not measured here, this reduction in oncology visits may improve assessment and treatment times for non-LC patients, being assessed by MDC clinicians who treat other cancer subtypes.

There was a trend towards a reduction in the mean number of biopsies and staging imaging completed, which has previously been demonstrated by Voong et al. [3]. This possible reduction in health resource utilization has wider impacts, including avoiding exposing patients to unnecessary morbidity (related to radiation and biopsies), reducing patient travel for tests, and reducing resource wait times for other non-LC patients. This warrants further evaluation in larger-scale studies.

Guideline-recommended mediastinal staging increased significantly in the MDC (15.0 to 60.0%). Friedman et al. found a similar significant increase in mediastinal staging for suspected stage 3 NSCLC [45] in MDC (from 24.5 to 57.7%), although they did not include patients with stage 2 NSCLC. This increase in adherence to guideline-recommended care for stage 2/3 LC speaks to improved physician communication and a better understanding of each other’s roles in helping to develop treatment plans in MDC [14]. The MDC likely also allowed oncologists to relay more immediately their perception of the importance of mediastinal staging in developing a treatment plan to the Respirologist(s) in MDC, and likely has had a long-term impact in Respirologist practice.

There was no difference in patients receiving curative intent therapy in the MDC. Several studies have examined the rates of curative intent therapy in MDC, with mixed results. Ray et al. found non-significant increases in chemoradiotherapy for patients with stage 3 NSCLC [15] while Martin-Ucar et al. found increased rates of surgical resection in the MDC model [53]. These mixed results speak to the heterogeneity of LC patients, even when analysed by stage and pathologic subtype, and likely reflect nuances in multidisciplinary decision-making processes, and variability in MDC formats.

This study also demonstrates several unanticipated benefits, including expediting the care for patients with symptomatic metastatic disease and providing concurrent expedited care for patients with a non-LC diagnosis. We facilitated care for 31 patients with symptomatic metastatic disease requiring urgent assessment and/or treatment. While it is difficult to determine whether this led to any health system cost savings, timely care and identification of patients at high risk for unplanned acute care admission has been associated with reduced hospitalizations [54]. Furthermore, patients with non-lung cancer subtypes also benefitted from MDC, receiving concurrent oncology assessment when the MO or RO in clinic also treated that corresponding non-LC disease site. This has not previously been demonstrated in other MDC clinics and warrants further exploration as to the associated timeliness of care improvements for these patients. There were five patients in MDC who ultimately were found to have benign lesions; notably, despite early oncology consultation, none of these patients actually received therapy inappropriately. While we cannot compare this data to our baseline (as this data is not available), we previously demonstrated no significant change in the percentage of patients who were referred to and assessed by oncology [18]. This suggests that the development of MDC did not increase the oncology referrals for benign lesions.

MDC led a total out-of-pocket cost savings to be CAD 24,167 (CAD 69/patient) related to a reduction in travel and parking costs associated with clinic visits. Not surprisingly, this benefit was observed by patients; in fact, a qualitative study evaluating patient perceptions of LC care in the MDC model revealed that patients receiving care in the MDC reported convenience of multiple same-day assessments and the positive effects on the patient experience and availability of family support [14]. While it is challenging to measure the overall impact of increased family presence in the consolidated appointment structure, many studies have shown the value of improved caregiver involvement [12]. While several studies descriptively outline these out-of-pocket costs [4,5,12], Wood et al. quantified patient costs to be EUR 848 (~1290 CAD), which included child care, wages forgone, and transportation costs [32]. We did not capture childcare costs. Lastly, caregiver attendance likely yields unmeasurable impacts, by ensuring patients understand their diagnosis, providing psychosocial support, and helping with health system navigation [55,56].

From a societal perspective, LC care poses a significant burden, with losses estimated in the billions annually [10,57,58]. We found that there was a total reduction of CAD 6379 in patient productivity losses and CAD 17,335 in caregiver productivity losses in MDC, assuming a caregiver employment rate of 50%. While there have not been studies in a Canadian context for comparison, Wood et al. found the annualized productivity loss attributable to NSCLC to be EUR 1,484 (~CAD 2265) for patients and EUR 2839 (~CAD 4330) for caregivers [32] while Yabroff et al. found that informal caregiver costs over a 2-year period were USD 72,702 (~CAD 102,500) [37]. These costs often garnish limited attention but represent a significant personal society economic burden [37].

With respect to costs associated with clinic visits, the MDC resulted in cost savings of CAD 508 related to the reduced administrative burden. Several studies have measured these impacts [3,4,5,12], with one study estimating that MDC reduced health system cost per patient by 23% [3] (or CAD 5839 savings/patient); however, this analysis accounted for system costs not included in our analysis. Lastly, we did not look at health system cost categories that were common to both MDC and the traditional model of care in our analysis, such as the number of physician consults between referral and treatment, which previously demonstrated this to be unchanged between the two models; as such, physician billings would be unchanged between models and are therefore not reported [18].

The increase in EBUS uptake paradoxically resulted in increased health system and patient costs, including an additional CAD 98,563 in the MDC model. However, from the threshold analysis, we found that the increased use of invasive mediastinal staging observed in our study cost CAD 25,707 per QALY, well below previously published willingness to pay thresholds (CAD 80,000) [24]. Furthermore, improved adherence to guideline-recommend staging is likely to improve patient outcomes through reduced surgical mediastinal staging, reduced treatment burdens, and cancer down-staging [59].

### Study Limitations

This is a single centre study in a public hospital system; however, the study demonstrates important benefits of continuous QI in LC care. While we previously demonstrated improved timeliness of assessment and treatment, as well as patient satisfaction in MDC, we have not yet studied patient survival, as this was not the intention of our initiative, and this data has not yet matured to perform this analysis. Furthermore, assessment of mortality can be confounded by multiple other variables, such newer treatments (immunotherapy, checkpoint inhibitors, etc.) for LC. Although there was an increase mediastinal staging in MDC, it is possible that reflects the evolution and increased uptake of EBUS at KHSC, as our local EBUS program only started in 2014. We also acknowledge the 8th Edition LC staging guidelines were released during our study (1 January 2017), however nodal staging was unchanged [60].

Furthermore, retrospective cost analysis is challenging where there is continuous clinic refinement and a mix funding for care. However, we conducted sensitivity analyses demonstrating that parameter uncertainty did not substantially affect the results. There may ultimately be unaccounted for random sampling error arising from the study sample size. The lack of visit standardization at KHSC Cancer Centre (i.e., number and type of nursing assessments and triage) made assessing resource utilization difficult. That said, registration clerk resources are standard across the site. Finally, while there are limitations to extrapolating cost savings, particularly in terms of impact of sample size, presenting summative cost savings help to understand the overall potential impact of an initiative according to scale.

Lastly, while we did not measure the duration of clinic visits directly, and thus relied upon published clinic visit durations in our sensitivity analysis, a qualitative thematic analysis of the MDC clinic published by our group identified that physicians noted increased efficiency of use of clinic time, enabling assessment of a large number of patients per clinic [14]. As such, clinic visit durations would likely be similar or shorter to that used in the current analyses.

It is difficult to account for societal cost reductions fully in our study; however, our qualitative study captured some of these themes anecdotally [14]. We acknowledge the potential error in our economic and sensitivity analysis. Productivity loss of 50% in our base model reflects previous literature and clinical observations of caregiver age [10,32,33]. We observed clinically that the age status of caregivers roughly reflects that of the patients they accompany (23% < 65 years old) with many having an adult child present at the visit as well, leading us to believe that 50% loss of caregiver productivity is appropriate for the cohort as a whole. The cost savings per patient likely represent an underestimate, given other unmeasured out-of-pocket costs (childcare, meals, etc.). Lastly, We acknowledge that the costs included in the invasive mediastinal staging sensitivity analysis may vary depending on the costing source, healthcare system, and types of costs included (i.e., procedural sedation vs. general anaesthesia) [24], however we chose the costing reference that most closely resembles our model with EBUS-TBNA provided under conscious sedation, within the same single-payer healthcare system and province [38].

While we recognize an imbalanced sample size of patients in the traditional model (78 patients) compared with the MDC model (350 patients), the baseline period was selected in accordance with recommendations for assessing baseline stability in a process from which variation can be assessed [61]. The sample size of the traditional model was chosen for our initial QI study [18], from which we continued to measure prospectively for further improvements. Despite this, the large number of patients studied in this MDC model represents a relative strength of our study, as the sample size is larger than many prior studies [11].

Finally, we acknowledge the paucity of Thoracic Surgery presence in the MDC during the period of evaluation. As mentioned previously, at our institution, Respirology and Thoracic Surgery run parallel LC assessment clinics, to which patients are triaged according to suspicion of resectable and operable LC based on available clinical information and imaging at the time of triage. The quality improvement initiative upon which this study is based sought to streamline care in the larger Respirology clinic and represents the focus of the present evaluation; however, ongoing improvement efforts are underway to increased participation of Thoracic Surgery in the MDC, which is likely to further reduce patient out-of-pocket expenses through streamlined care.

## 5. Conclusions

Continuous QI of an LC MDC has led to increased clinic capacity and standardization, a significant reduction in individual oncology visits prior to treatment, and both patient and health system cost savings. Unanticipated secondary benefits included facilitating oncology assessment for patients with a non-LC diagnosis and symptomatic metastatic disease. There was a significant increase in guideline-recommended mediastinal staging for patients with stage 2 or 3 disease, for which despite an increase in costs associated with the procedure, was still cost effective. When analysed over 350 patients, the MDC model resulted in a total savings of CAD 48,389 in out-of-pocket, societal, and clinic visit costs over 22 months, for a per patient savings of CAD 138. Extrapolating this cost savings to an estimated 585 patients seen in the MDC over the first 3 years of the program, cost savings to date are estimated at CAD 80,882.10. This study highlights that continuous QI can lead to patient and health systems benefits and significant cost savings.

## Figures and Tables

**Table 1:** Patient demographics in the traditional and multidisciplinary clinic models.

Variable	Traditional Model (*n* = 78)	MDC (*n* = 350)
Patient Characteristics		
Mean Age in Years (± SD)	70.0 (9.7)	71.7 (9.1)
Pathologic LC Diagnosis		
NSCLC	69 (88.5)	260 (74.3)
SCLC	9 (11.5)	45 (12.9)
Presumed LC	N/A	45 (12.9)
Clinical LC Stage		
1	24 (30.8)	109 (31.1)
2	6 (7.7)	26 (7.4)
3	14 (18.0)	69 (19.7)
4	29 (37.3)	143 (40.9)
Undetermined	5 (6.4)	3 (0.9)
Mean Return Distance to KHSC (km)	N/A	102.0
**Diagnosis of Patients with Non-LC Pathology that Received Consultation in MDC (*n* = 24)**		
Cancer Type		
Breast	N/A	8
Mesothelioma	N/A	4
Other Non-LC	N/A	12

Data presented as *n* (%) unless otherwise specified. Abbreviations: KHSC, Kingston Health Sciences Centre; LC, Lung Cancer; MDC, Multidisciplinary LC Clinic; NSCLC, Non-Small Cell Lung Cancer; NOS, Not Otherwise Specified; RT, Radiation Therapy; SCLC, Small Cell Lung Cancer; SD, Standard Deviation; ST, Systemic Therapy; TS, Thoracic Surgery.

**Table 2:** Oncology visits and health resource utilization during the diagnosis to treatment time interval in the traditional and multidisciplinary clinic models.

	Traditional Model	MDC	*p* Value
Mean Oncology Visits per Patient from Diagnosis to Treatment ^a^			
All patients	2.68	1.62	<0.001
Stage 1 LC (*n* = 133)	2.29	1.66	0.006
Stage 2 LC (*n* = 32)	2.33	2.13	0.58
Stage 3 LC (*n* = 83)	3.43	2.03	0.004
Stage 4 LC (*n* = 172)	2.55	1.29	<0.001
**Health Resource Utilization from Diagnosis to Treatment**			
Mean Biopsies per Patient	1.32	1.17	0.18
Mean Staging Imaging per Patient	2.24	2.02	0.20
Invasive Mediastinal Staging by EBUS-TBNA for patients with stage 2 or 3 LC % (*n*)	15.0% (3/20)	60.0% (57/95)	<0.001
**Treatments Received by LC Patients ^a^**			
Stage 1 LC curative TS %(*n*)	20.8% (5/24)	13.7% (15/109)	0.44
Stage 1 LC curative RT %(*n*)	87.5% (21/24)	77.1% (84/109)	0.20
Stage 2 LC curative TS %(*n*)	33.3% (2/6)	23.1% (6/26)	0.67
Stage 2 LC curative RT %(*n*)	83.3% (5/6)	65.4% (17/26)	0.37
Stage 3 ST + RT %(*n*)	57.1% (8/14)	53.6% (37/69)	0.82
Stage 4 LC receiving ST %(*n*)	37.9% (11/29)	39.9% (57/143)	0.85

Abbreviations: EBUS-TBNA, Endobronchial Ultrasound Transbronchial Needle Aspiration; LC, Lung Cancer; MDC, Multidisciplinary LC Clinic; NSCLC, Non-Small Cell Lung Cancer RT, Radiation Therapy; ST, Systemic Therapy; TS, Thoracic Surgery. ^a^ Patients with undetermined LC stage (5 traditional models and 3 MDC) were not included in visits and treatment subgroup analyses.

**Table 3:** Economic benefits of MDC versus traditional model of care.

Variable	Base Case
Patient Visits Saved [*n* = 350]	371
Caregiver Visits Saved [1.25 caregivers/patient visit saved]	464
Patients <65 years of age	23%
Parking Cost	$6.00/visit
Clinic Visit Duration	1.26 h
Time Forgone (return travel, parking, finding clinic)	1.27 h
Caregivers Incurring Productivity Loss	50%
**Out-of-Pocket Cost Savings**	
Parking Cost ($6/visit)	$2226
Return Travel Cost ($59.14/visit)	$21,941
Out-of-Pocket Cost Savings per Patient	$69
**Total Out-of-Pocket Cost Savings**	$24,167
**Productivity Loss Savings**	
Patient Opportunity Cost ($29.55/hour)	$6379
Caregiver Opportunity Cost ($29.55/hour)	$17,335
**Total Productivity Loss Savings**	$23,714
**Total Patient Out-of-Pocket Expenses + Productivity Loss Savings**	$47,882
**Clinic Visit Cost**	
Cost for Time Spent Booking Appointments ($1.37/visit)	$508
**Total Personnel Cost Savings**	$508
**Total Savings**	$48,389

Abbreviations: $, 2019 CAD.

**Table 4:** Threshold analysis for invasive mediastinal staging by EBUS-TBNA.

Unit Variables		
EBUS-TBNA Cost	$1577/procedure	
Caregiver Visits [1 caregivers/EBUS-TBNA Procedure]	3	
Patients <65 years of age	33%	
Parking Cost	$9.00/visit	
Time Forgone (return travel, parking, procedure, recovery)	7.5 h	
Caregivers Incurring Productivity Loss	50%	
Quality-Adjusted Life Years/EBUS-TBNA	0.071	
**Threshold Analysis**	**Traditional Model**	**MDC**
# EBUS-TBNA Procedures	3	57
Quality-Adjusted Life Years	0.21	4.05
**Procedural Costs**		
EBUS-TBNA	$4731	$89,889
**Out-of-Pocket Costs**		
Parking Cost ($9/visit)	$14	$257
Return Travel Cost ($59.14/visit)	$177	$3371
**Total Out-of-Pocket Costs**	$192	$3627
**Productivity Loss Costs**		
Patient Opportunity Cost ($29.55/hour)	$221	$4207
Caregiver Opportunity Cost ($29.55/hour)	$333	$6316
**Total Productivity Loss Savings**	$554	$10,523
**Total EBUS-TBNA Cost**	$5476	$104,039
**Cost per QALY**	$25,708	

Abbreviations: EBUS-TBNA, Endobronchial Ultrasound Transbronchial Needle Aspiration; QALY, Quality-Adjusted Life Years; $, 2019 CAD.

## Data Availability

The data presented in this study are available on request from the corresponding author. The data are not publicly available due to confidentiality concerns.

## Supplementary Materials

The following are available online at https://www.mdpi.com/article/10.3390/curroncol28030157/s1, Table S1. Variable Definitions, Table S2. Cost and Time Estimates per Oncology visit. Table S3: Economic benefits of MDC versus traditional model of care, sensitivity analysis. Table S4. Cost and Time Estimates per EBUS-TBNA Procedure.
