# Supplementary material for: Health Resource and Cost Savings Achieved in a Multidisciplinary Lung Cancer Clinic
Source: Curr Oncol. 2021 Apr 29;28(3):1681–95. doi: 10.3390/curroncol28030157 (PMC8161784; doi:10.3390/curroncol28030157)
Supplement: Supplementary file 1 [file curroncol-28-00157-s001.zip › curroncol-1180383-supplementary.pdf]

Table S1. Variable Definitions.

Cost Category	Definition
Travel Distance	Round trip from patient's home to Hotel Dieu Hospital, Kingston, Ontario
**Patient Expenses**	
Travel Cost	Cost per kilometre
Travel Time	Distance travelled/speed of travel
Wage	Average hourly wage in 2019 for Canadians over the age of 25
Oncology Clinic Appointment Duration	Registration time and physician meeting duration
Time Spent Locating Clinic	Walking to clinic from parking location
Productivity Loss	Average Oncology Clinic Appointment Duration + Time Spent Locating Clinic + Parking Time + Travel Time
Patient Cost of Productivity Loss	Hours of productivity lost multiplied by wage, excluding any person over the age of 65
Caregiver Cost of Productivity Loss	Hours of productivity lost multiplied by wage, assuming employment of 50% (25–75%) of caregivers present
Parking Time	Average Oncology Clinic Appointment Duration + Time Spent Locating Clinic
Parking Cost	Cost per hour, average appointment duration and time spent locating the clinic
Total Patient Expenses (<65 years old)	Travel Cost + Cost of Productivity Loss + Parking Cost
Total Patient Expenses (>65 years old)	Travel Cost + Parking Cost
**Clinic Visit Expenses**	
Administrative Assistant Base Wage	Average hourly wage in 2019 for administrative assistants
Administrative Assistant Benefits	Benefits
Administrative Assistant Total Wage	Hourly base wage + benefits
Appointment Booking Time	Time to book one appointment for one patient
Cost Per Patient	The cost of booking one appointment for one patient

Table S2. Cost and Time Estimates per Oncology visit.

Variable	Value	Notes
Patient Visits Saved	371	(2.69–1.62) X 350 patients
Caregivers Visits Saved	464	371 patient visits X 1.25 caregivers/patients/visit
Return Travel Distance	101.96 km	Calculated mean two-way distance per patient using available data
Caregiver Productivity Loss	50%	50% in base case, 25% best case, 75% worst case
**Out of Pocket and Productivity Expenses**		
Travel Cost	$0.58/km	$0.58/km: $0.58 X 101.96 km = $59.14 per visit<br>Based on 2019 automobile allowance rates[26]
Travel Time	61.2 min	Estimated: 100km/h average speed and mean distance of 101.96 km
Wage Loss	$29.55/hour	2019 average hourly wage for Canadians over 25[28]
Oncology Clinic Visit Duration	75.60 min	As per Gao et al.[29]
Time Spent Locating Clinic	15 min	Estimated
Total Time Lost Per Visit	2.53 h	Average Oncology Clinic Appointment Duration (75.6 min) + Time Spent Locating Clinic (15 min) + Travel Time (61.2 min)

Cost of Productivity Loss per Visit	$74.76	Productivity loss at a rate of $29.55/hr for 2.53 h of lost time
Parking Cost	$6.00	KHSC parking rate, $3.00/hr X 2 h parking/average visit
**Clinic Visit Expenses**		
Administrative Assistant Base Wage	$21.85/hour	Wage range $20.25-$23.47; average used
Administrative Assistant Benefits	$5.47	25% of base wage
Administrative Assistant Total Wage	$27.33/hour	Average Base wage ($21.85) + Benefits ($5.47)
Appointment Booking Time	3 min	Estimated time taken to book one appointment using available data
Cost Per Appointment Booking	$1.37	Calculated using hourly total wage and 3 min taken to book appointment

Abbreviations: $, 2019 CAD.

Table S3: Economic benefits of MDC versus traditional model of care, sensitivity analysis**.**

Variable	Best Case	Worst Case
Patient Visits Saved [$n = 350$]	371	371
Caregiver Visits Saved [1.25 caregivers/patient visit]	464	464
Patients <65 years of age	23%	23%
Parking Cost	$4.50/visit	$12/visit
Clinic Visit Duration	0.93 h	1.58 h
Time Forgone (return travel, parking, finding clinic)	1.26 h	5.92 h
Caregivers Incurring Productivity Loss	25%	75%
**Out-of-Pocket Cost Savings**		
Parking Cost (cost/visit)	$1,670	$4,452
Return Travel Cost ($59.14/visit)	$21,941	$21,941
**Total Out-of-Pocket Cost Savings**	$23,610	$26,393
**Productivity Loss Savings**		
Patient Opportunity Cost ($29.55/hour)	$5,535	$18,911
Caregiver Opportunity Cost ($29.55/hour)	$7,520	$77,084
**Total Productivity Loss Savings**	$13,055	$95,995
**Total Patient Out-of-Pocket Expenses + Productivity Loss Savings**	$36,665	$122,388
**Clinic Visit Cost**		
Cost for Time Spent Booking Appointments ($1.37/visit)	$508	$508
**Total Personnel Cost Savings**	$508	$508
**Total Savings**	$37,173	$122,896

Abbreviations: $, 2019 CAD.

Table S4. Cost and Time Estimates per EBUS-TBNA Procedure.

Variable	Value	Notes
Return Travel Distance	101.96 km	Calculated mean two-way distance per patient using available data
Caregiver Productivity Loss	50%	50%
**EBUS-TBNA, Out of Pocket and Productivity Expenses**		

EBUS-TBNA Procedure Cost	$1,577*	Per Czarnecka-Kujawa et al.[38]
Travel Cost	$0.58/km	$0.58/km: $0.58 X 101.96 km = $59.14 per visit<br>Based on 2019 automobile allowance rates[26]
Travel Time	61.2 min	Estimated: 100 km/h average speed and mean distance of 101.96 km
Wage Loss	$29.55/hour	2019 average hourly wage for Canadians over 25[28]
EBUS-TBNA Duration	180 min	(Includes patient registration, preparation, and recovery)
Total Time Lost Per EBUS-TBNA Visit	7.5 h	We consider time lost to EBUS-TBNA Visit to be a full work day as patients are advised not to work after the procedure, and most patients take a full day off
Cost of Productivity Loss per Visit	$221.40	Productivity loss at a rate of $29.55/hr for 7.5 h of lost time
Parking Cost	$9.00	KHSC parking rate, $3.00/hr X 3 h parking/average visit for an EBUS

* CAD 1,468 (2015) = CAD 1,577 (2019)
Abbreviations: $, 2019 CAD.
